# Adolescent alcohol and nicotine exposure alters the adult response to alcohol use

**DOI:** 10.3389/adar.2023.11880

**Published:** 2023-11-22

**Authors:** Sheketha R. Hauser, Robert A. Waeiss, Gerald A. Deehan, Eric A. Engleman, Richard L. Bell, Zachary A. Rodd

**Affiliations:** ^1^ Department of Psychiatry, Indiana University School of Medicine, Indianapolis, IN, United States; ^2^ Stark Neurosciences Research Institute, Indiana University School of Medicine, Indianapolis, IN, United States; ^3^ Department of Psychology, East Tennessee State University, Johnson City, TN, United States

**Keywords:** adolescence, alcohol, nicotine, cholinergic system, co-abuse

## Abstract

Adolescence through young adulthood is a unique period of neuronal development and maturation. Numerous agents can alter this process, resulting in long-term neurological and biological consequences. In the clinical literature, it is frequently reported that adolescent alcohol consumption increases the propensity to develop addictions, including alcohol use disorder (AUD), during adulthood. A general limitation of both clinical and human pre-clinical adolescent alcohol research is the high rate of co-using/abusing more than one drug during adolescence, such as co-using/abusing alcohol with nicotine. A primary goal of basic research is elucidating neuroadaptations produced by adolescent alcohol exposure/consumption that promote alcohol and other drug self-administration in adulthood. The long-term goal is to develop pharmacotherapeutics for the prevention or amelioration of these neuroadaptations. This review will focus on studies that have examined the effects of adolescent alcohol and nicotine exposure on adult alcohol consumption, the hypersensitivity of the mesolimbic dopaminergic system, and enhanced responses not only to alcohol but also to nicotine during adulthood. Again, the long-term goal is to identify potential cholinergic agents to prevent or ameliorate the consequences of, peri-adolescent alcohol abuse.

## Human research examining adolescent alcohol consumption

Adolescence is a period in which humans begin to use illicit and age-restricted drugs. Although adolescent drinking has decreased from 2002 to 2021, there is still a high prevalence of adolescent alcohol drinking in the United States [1]. This includes 58% of 12th graders reporting the use of alcohol within the past year and 28% of which engaged in binge drinking within the previous 2 weeks (i.e., >5 or more consecutive drinks per drinking episode) [2, 3]. Binge drinking is defined by the National Institute on Alcohol Abuse and Alcoholism as 4+/5+ drinks for women/men per occasion respectively (achieving blood alcohol concentrations (BACs) of 0.08 g/dL = 80 mg/dL) [4]. Binge drinking is exaggerated in US young adults since the average binge episode consists of 9.5 drinks/occasion [5, 6].

A recent trend in adolescent/young adult drinking is that the initiation of binge drinking has become progressively earlier, and a sharp increase in the overall rate of binge drinking during the transition from late adolescent/young adulthood into adulthood [7]. Moreover, there has been a focus on studying the effects of high-intensity and extreme-intensity binge drinking in adolescents [8–10]. A longitudinal study comparing US alcohol consumption from 2005 to 2015 indicated consistent levels of binge drinking in 18 year-olds (approximately 20%) [11]. There were also significant rates of high- (approximately 10%) and extreme-intensity (approximately 5%) binge drinking among these US 18 year-olds [11]. In young adults, recent data have indicated that roughly 30% report binge drinking, 11% report high-intensity binge drinking, and 5% report extreme-intensity binge drinking [11].

Alcohol consumption during adolescence is associated with several deleterious consequences. For example, adolescents and young adults display, relative to their adult counterparts, heavier drinking bouts, arrests for driving with ability impaired, and an increased number of arrests for driving while impaired, and an increased rate of alcohol dependence during adulthood significantly associated with the age of the first alcohol drink and the number of binge alcohol drinking episodes during adolescence [12, 13]. The adolescent brain appears to be more susceptible to the effects of binge alcohol consumption than the adult brain [14, 15], and neuroadaptations produced by adolescent alcohol drinking are thought to underlie an increased rate of alcohol misuse in adults suffering from AUDs [16].

Epidemiological studies indicate that individuals who initiate alcohol use before age 15 are 1.3–1.6 times more likely to suffer from an AUD [17]. Additionally, having a family history that is positive [positive (FHP)] for an AUD combined with the initiation of alcohol consumption during adolescence dramatically increases the risk of adult alcohol dependence [18, 19]. A family history of alcoholism also significantly increases observed alterations in white matter integrity (fractional anisotropy) of adolescent binge-drinking subjects [19]. Overall, the clinical evidence consistently indicates that alterations in the central nervous system (CNS) produced by adolescent alcohol consumption are enhanced in individuals with a family history of alcoholism.

A major caveat concerning the interpretation of clinical data on the influence of adolescent alcohol consumption on adult alcohol consumption and the development of an AUD, was the difficulty of adult subjects in recalling their alcohol consumption during adolescence. Given long-term alcohol use and misuse can dramatically affect memory and recall, the accuracy of such first-person accounts have been called into question [20]. However, several recent longitudinal studies have provided clinical support that adolescent alcohol consumption increases the likelihood of adult alcohol consumption or AUD. In a Swedish military conscript study, young adults consuming high-intensity levels of alcohol (8.6 g/day) displayed an increase in alcohol consumption during later adulthood and developed higher rates of developing an AUD [21]. Specifically, the total consumed dose of alcohol during young adulthood increased the future risk for developing an AUD, but a pattern of heavy episodic drinking (high-intensity drinking) significantly increased the later risks for developing an AUD and cirrhosis of the liver [21]. A parallel longitudinal study examining Norwegian and Australian adolescents [22] reported that adolescent alcohol drinking was associated with an increase in adult alcohol consumption and the rate for developing an AUD. Furthermore, the study indicated that interfering with early adolescent alcohol consumption has a protective effect on drinking patterns during late adolescence and adulthood [22]. Overall, recent longitudinal studies have replicated the initial longitudinal study’s findings on the deleterious effect of adolescent alcohol consumption on the rate of AUD during adulthood [23–25].

## Adolescent and adult alcohol consumption in rodents

### The effect of voluntary alcohol consumption during adolescence on adult alcohol consumption

Pre-clinical studies use rodent models to investigate the effect of adolescent alcohol (ethanol) exposure on subsequent neurobiological alterations that contribute to observed changes in behavior related to alcohol drinking in adulthood. The animals are typically exposed to alcohol between early adolescence to late adolescence–emerging adulthood (i.e., postnatal day (PND) 28–65; Table 1), which corresponds with 13–25 years of age in humans [14, 26, 27].

**TABLE 1:** Rat and human ages.

Rat ages [Post-Natal Days (PNDs)]										
PNDs 1–7	PNDs 8–21	PND 21	18–22	22–27	28–42	43–60	61–75	76–90	90+
3rd Trimester	Infant	Weaning	Childhood	Juvenile	Adolescence	Peri-Adolescence	Emerging Adult	Young Adult	Adult
Human Ages (Years)										
−0.25-0.0 Years	0–2 Years	0.5–2 Years	2 to 6	7–12	13–18	18–21	21–24	25–28	28–50s
					Puberty					
							Adapted from (Bell et al., 2014)			

Overall, the field has developed several paradigms to assess rodent drinking, expose animals to binge-like levels of ethanol intake, and/or establish the level of motivation to self-administer ethanol (i.e., operant responding/behavior to obtain alcohol). In typical voluntary free-choice models, animals are allowed access to 2- or 3- bottles with a choice between ethanol or water, or multiple solutions, and animals can freely consume fluid over a 24 h period. Using a slightly different approach, an intermittent drinking paradigm involves alternating periods of access to alcohol with periods of imposed abstinence to establish binge-like intake in outbred rodents [28]. Studies measuring voluntary ethanol consumption in adolescent non-selected (outbred) rats have produced variable findings. There have been reports that voluntary ethanol drinking during adolescence (PND 28 or PND 31) and continued into adulthood (PND 69–70 or PND 71) had no effect on adult ethanol consumption [29, 30]. On the other hand, Amodeo et al. [31] found that adolescent animals (PND 26–59) exposed to an intermittent voluntary ethanol paradigm, during a period of social isolation, exhibited an increase in operant “appetitive” but not “consummatory” behavior related to ethanol intake during adulthood. Interestingly, after separating the adolescent rats into low (average of 0.29 g/kg/30 min exposure) and high (average of 0.65 g/kg) voluntary ethanol consumption during adolescence, there was a significant increase in adult consumption of ethanol in the ‘high’ adolescent ethanol drinkers [31].

Adulteration of an ethanol solution using a sweetener (i.e., sucrose or saccharin) enhanced voluntary ethanol consumption during adolescence (PND 29–54; PND 28–42, respectively) [32, 33] and produced biologically relevant blood ethanol concentrations (BEC) as well as enhanced consumption of the same sweetened ethanol solution in adult non-selected rats, respectively. However, this is not always the case. Gilpin et al. [34] reported that operant voluntary binge ethanol consumption in adolescence (PND 28–42) resulted in pharmacologically relevant binge blood ethanol concentrations BAC levels (≥80 mg/dL); however, operant voluntary binge ethanol consumption in adolescence did not alter adult operant self-administration of sweetened ethanol solution or unsweetened ethanol solution in male Wistar rats.

Adolescent voluntary drinking studies using mice have also reported mixed findings on subsequent adult ethanol drinking. An early study using C57BL/6J mice found that adolescent ethanol consumption (beginning at weaning: 3 weeks of age; PND∼21) was associated with increased ethanol consumption in adulthood [35]. Nevertheless, voluntary ethanol consumption starting at 5 weeks of age (PND 35) in BALB/cByJ mice also exhibited a greater ethanol preference in adulthood [36]. In contrast, other studies did not observe the same increase in adult consumption following adolescent ethanol exposure (starting at ∼PND 21 or ∼PND 35) in C57BL/6J [37].

Studies utilizing the murine model have demonstrated that “intermittent-type access to ethanol during adolescence can produce pharmacologically relevant BECs and facilitate adult ethanol intake, but the findings seem to be strain dependent [38, 39]. For example, utilizing the scheduled high ethanol consumption (SHAC) [40] binge procedure produces adolescent BECs of >80 mg/dL in male and female C57BL/6J mice [38]. Moreover, these authors reported that adolescent females were more vulnerable to, i.e., displayed more of, this effect than males [38]. Researchers using the drinking-in-the-dark (DID) binge drinking model, where animals have access to ethanol for 2 or 4 h and 3 or 4 h into the dark cycle, have also reported that adolescent ethanol exposure (PND 28–42) in C57BL/6J, mice produced significantly higher ethanol consumption in adulthood, a finding that was not observed DBA/2J mouse strain [41]. Adult ethanol consumption was also enhanced in C57BL/6J mice following adolescent access [PND 28–36 to low concentration of ethanol (5%; 20 mg/dL BEC) during a modified DID exposure, whereas adolescent DID ethanol consumption (20%, 20 mg/dL BEC)] in DBA/2J did not alter ethanol consumption in adulthood [42]. These findings would suggest that, while the DID paradigm can produce binge-like ethanol intake in adolescent animals, genetic background likely plays a role as well. That is, genetic factors that have established the C57BL/6J mouse line as a high ethanol-drinking model, likely contributed to the observed increase in ethanol intake in adulthood as the effect was present even when adolescent animals were exposed to low concentrations (non-binge levels: BEC <80 mg/dL) [42].

Collectively, the literature suggests that under certain conditions, voluntary ethanol consumption during adolescence can produce enhanced ethanol consumption in adulthood. In some cases, alterations in drinking behavior, due to adolescent ethanol exposure, may be mediated by sex-of-animal effect and/or genotype, however, obtaining biologically relevant blood ethanol levels, (BEC >80 mg/dL) appears to be a critical factor. Towner and Varlinskaya [39] reported that one-third of voluntary adolescent ethanol consumption in rodents resulted in a subsequent increase in adult ethanol consumption and that pharmacological levels of ethanol during adolescence may have to be well above a BEC of 80 mg/dL (e.g., 100–200 mg/dL) to increase subsequent adult ethanol consumption. Thus, DID, SHAC, and forced ethanol (e.g., IP, IG, vapor inhalation) procedures in adolescent rodents tend to result in higher BECs and more consistent increases in adult ethanol consumption [39].

## Voluntary alcohol consumption during adolescence effects on adult alcohol consumption in alcohol-preferring rodent models

Selective breeding for high ethanol intake has produced multiple rat lines that voluntarily consume pharmacologically relevant levels of ethanol under 24 h free-access drinking conditions [e.g., alcohol-preferring (P) and Alko Alcohol (AA)] [c.f. 43, 44]. Such lines have been utilized as a powerful tool to examine the influence of genetic background contributing to the behavioral and neurobiological components of AUDs.

### Alcohol preferring (P) rat line

The P rat line has demonstrated reliable consumption of biologically relevant levels of ethanol from post-natal day (PND) 7 until death) [45]. Twenty-four free-access drinking access during adolescence results in binge-level of ethanol intake (>80 mg%) in P rats [8]. In P rats, voluntary ethanol consumption during adolescence can alter ethanol-related behaviors during adulthood [46–49]. For example, 24 h free-access ethanol drinking during PND 22–71 increased ethanol consumption during ethanol re-exposure in adulthood (PND 99) compared to control animals [47]. Adolescent (PND 28–60) voluntary ethanol consumption in P rats (average intake of 6.3 g/kg/day) has also been shown to increase the rate of acquisition of operant ethanol self-administration during adulthood (PND 90) compared to animals exposed to the same regimen completely in adulthood (PND 137–169; testing started at PND 199) [48, 49]. Ethanol consumption during adolescence produced resistance to extinction, increased the expression of both context- and ethanol primed-induced ethanol seeking, enhanced relapse drinking, and significantly increased breakpoint in adulthood [48, 50]. Moreover, these effects on operant behavior were observed during the second cycle of testing for extinction, seeking, and relapse, suggesting that voluntary adolescent ethanol consumption can produce persistent effects on ethanol-related behaviors well into adulthood [48]. It is also important to mention that these findings were specific to ethanol as a control study using the same paradigm, but exposing animals instead to saccharin during adolescence, did not alter adult saccharin self-administration, saccharin extinction learning, relapse, or breakpoint [50].

### Alko, alcohol (AA) rat line

The AA rat line also readily consumes pharmacologically relevant levels of ethanol (5–8 g/kg) in 24 h with BECs as high as 50 mg% [51–53]. A recent report found that voluntary adolescent ethanol consumption (starting at PND 42 and continuing for 6 weeks) in female AA rats did not increase subsequent ethanol drinking in adulthood, and by extension did not increase ethanol preference [54]. Although the AA rats consumed pharmacologically relevant levels of ethanol during adolescence, animals failed to establish binge-like (>80 mg%) BECs, which may have been a reason as to why the authors failed to observe increased ethanol consumption in adulthood [54]. Further, the timing of adolescent exposure (i.e., mid-late adolescence) may have also been another factor. Previous work suggests that early-mid (PND 28–45) adolescent exposure induces more severe neuroadaptations than mid-late ethanol exposure [26, 54]. Regardless, the data supports the notion that, even in alcohol-preferring rat models, attaining high (i.e., binge-like or higher) BECs during adolescence appears to be important for subsequent increases in adult ethanol drinking.

## Adolescent intermittent ethanol (AIE) exposure effects adult alcohol consumption

### Vapor exposure of alcohol in adolescence

The adolescent intermittent ethanol (AIE) exposure model is an experimenter-administered binge model (e.g., ethanol vapor, intragastric (IG), intraperitoneal (IP) injection), used to produce consistent binge BEC levels in rodents that do not readily consume ethanol [cf., 6]. The BEC levels achieved with AIE are approximately 160 mg% (i.e., 0.16 g/dL) or greater in rats and mice [55–60]. However, studies utilizing AIE exposure have reported mixed findings. High level AIE vapor exposure (PND 28–42) that established BECs in excess of 300 mg% produced an increase in operant responding for (maintenance) and a resistance to extinction of operant self-administration during adulthood (PND 65–90) in Long-Evans [61]. However, utilizing an identical AIE regimen and rat strain, Nentwig et al. [62] failed to observe similar AIE-induced alterations to adult 2-bottle choice voluntary ethanol consumption or operant self-administration behaviors. Studies examining AIE in mice are also mixed. For instance, AIE (PND 28–42) vapor exposure increased ethanol consumption in adult male C57BL/6J mice following short-term abstinence during late adolescence and early adulthood (PND 50–76) and following protracted abstinence (PND 70–97) in adulthood, but did not alter consumption in female mice [63] indicating that potential sex differences may be at play in vulnerability to the effects of AIE vapor exposure.

### Systemic administration of alcohol in adolescence

Several studies have utilized the systemic route of administration (i.e., IP injection or IG gavage) of ethanol during adolescence to examine the long-term effects on ethanol drinking in adulthood. Ethanol administered via the IP or IG routes has been shown to produce equivalent ethanol exposure between subjects that is not approximated in consumption/drinking paradigms, and reliable, dose-dependent, BECs. However, much like the research discussed thus far, the findings have been mixed and there seem to be dose- and sex-dependent, as well as age-related variables that underlie the enduring behavioral effects observed using these techniques.

An early study by Gilpin et al. [34] reported that adult animals exhibited a conditioned taste aversion to sweetened solutions and consumed significantly less sweetened and unsweetened ethanol following adolescent ethanol injections (PND 28–42; 2 g/kg/IP) compared to control animals. In contrast, administration of a lower dose of ethanol (1.5 g/kg/IP; PND 30–50) enhanced consumption of a sweetened ethanol solution during limited access testing in female adult rats (PND 65–80), whereas reducing the dose by half (0.75 g/kg/IP) did not [64]. A similar finding was reported using a higher dose of ethanol (3 g/kg/IP; PND 25–38) in that, adult animals (PND 60) exhibited an increase in ethanol consumption, which produced BECs that were not pharmacologically relevant (<30 mg%) [65]. In a similar experiment, ethanol administration of the same dose of ethanol (3 g/kg/IP) during early-mid adolescence (PND 30–43), but not late adolescence (PND 45–58), increased operant self-administration of ethanol as well as ethanol consumption when animals were provided both free and intermittent access ethanol in young adulthood [66].

Varying effects have also been reported in studies utilizing adolescent IG administration of ethanol. Maldonado-Devincci et al. [67] indicated IG administration of 1.5, 3.0, or 5.0 g/kg ethanol in 4 days intervals (PND 28–31, PND 35–38, and PND 43–45) increased ethanol consumption in young adulthood (PND 60–69) in both male and female Sprague-Dawley (SD) rats, an effect more prominent in male compared to female animals. Intermittent IG ethanol exposure (PND 28–48; 2 days on/2 days off) treatment in male and female P rats resulted in increased adult ethanol consumption (PND 90+) during both operant acquisition and relapse drinking conditions [68, 69]. In contrast, repeated gavage (every 8 h for 2 days: 6 treatments total) during adolescence (PND 30–32) decreased ethanol consumption in adulthood in Sprague Dawley rats [70]. The differences in behavioral data between these studies may be due to the gavage procedure, the length of time animals received exposure, and the strain of rats (P vs. SD rats).

## Adolescent alcohol consumption and nicotine use–clinical data

Co-use/abuse is common among adolescents and young adults [71, 72]. A limitation of research is the ability to account for co-use/abuse of more than one drug during the window of adolescence and the effect this may have on the development of drug-related issues later in life. Adolescent alcohol drinking has been linked to increased adulthood use of opioids, cannabis, nicotine, and other drugs of abuse [73, 74]. Specifically for nicotine, adolescent binge drinking enhances the likelihood of smoking during adolescence by 88% as well as during adulthood, while individuals who do not binge drink during this period have lower smoking rates during adolescence and adulthood [74, 75]. Moreover, adolescents who use nicotine have higher rates of AUDs than their non-smoking counterparts [75, 76]. Simultaneous alcohol and tobacco use during early adolescence (age: ∼12 years old) promoted an escalation of drug intake during late adolescence and was associated with a higher rate of adult drug addiction, AUD, and co-substance drug addiction [77].

The use of non-combustible nicotine via electronic delivery (i.e., electronic cigarettes [e-cigarettes] or vaping pens) has become a popular alternative to cigarette smoking, especially among adolescents over the last decade [78]. In 2016, 38% of all high school students stated they had tried e-cigarettes, a rate comparable to alcohol usage in the same demographic [79]. Initiation of e-cigarette use during adolescence is associated with a greater prevalence of traditional nicotine use later in life (cf. [6]) and may promote traditional nicotine use in individuals who would not have initiated use otherwise [80]. Adolescents who use e-cigarettes are 6.5 times more likely to consume alcohol (including bouts of binge drinking), compared to those who do not use e-cigarettes [81]. The liquid nicotine solutions for e-cigarettes can contain 92%–104% more nicotine than stated by the manufacturers (cf. [81]). In addition, individuals who “vape” also receive a significant dose of alcohol as the range of alcohol concentration in liquid nicotine solutions ranges from 0.4%–23.5%, with the most popular brands ranging from 10% to 18% [82]. The rate of absorption of alcohol through the “vaping’”route is extremely high, and alcohol metabolites can be detected in individuals actively “vaping” [82]. With “hacked” e-cigarette systems, the rate of alcohol, acetaldehyde, and aldehyde intake can increase in magnitude, and reach detectable levels within the brain without reaching detectable levels in the periphery [83]. Replicate findings have indicated that alcohol and nicotine co-use/abuse during adolescence enhances adult drug dependency compared to the consumption of only alcohol or nicotine during adolescence (cf. [84]).

## Modeling adolescent alcohol consumption and nicotine use–pre-clinical data

For several years, rodent experiments have sought to model ethanol/nicotine use/co-use during the developmental period of adolescence in the clinical population to determine the behavioral and neurobiological effects of these compounds that contribute to drug addiction both during adolescence and later in life. Several techniques have been used to investigate the effects of adolescent nicotine exposure on ethanol reward in adolescence and adulthood, such as oral nicotine access, injections (IP and subcutaneous: SC), intravenous (IV) self-administration, and nicotine vapor exposure. Overall, the findings are mixed as some rodent studies have demonstrated that adolescent nicotine exposure can increase ethanol consumption, while other studies report no effect. The discrepancy between studies seems to be related to when in adolescence (i.e., early, mid, or late) the animals are exposed to nicotine as well as the species used (mouse versus rat). For instance, oral nicotine exposure (200 μg/mL/22 h) during early-mid adolescence (∼PND 35–44) increases binge-like ethanol consumption and BECs in mid adolescence (∼PND 45–48) in female C57BL/6J mice [85, 86]. Oral nicotine consumption (30 μg/mL) using the DID model during late adolescence (PND 42–56) did not affect subsequent binge-like ethanol consumption in early adulthood in male C57BL/6J mice (PND 56–78) [87]. Repeated systemic administration of nicotine (0.4 mg/IP) during adolescence (PND 28–PND 42) induced long-lasting increases in adult ethanol self-administration, while adult (PND 60–74) nicotine administration did not alter subsequent adult ethanol self-administration in male Long-Evans rats [88].

Chronic continuous nicotine exposure (subcutaneous 21 day time-release pellets) during adolescence (PND 35–56) did not increase ethanol intake in SD rats during young adulthood (PND 53 -PND 74) [89]. Peripheral administration of nicotine (0.4 mg/kg/IP) administered in early adolescence (PND 28–32), prior to ethanol operant training, and then re-administered 2 h after ethanol training session during early-late adolescence (PND 33–56) did not alter self-administration or motivation (i.e., breakpoint) of sweetened ethanol [90]. Recently, Ruffolo et al. [91] examined the effects of vaporized JUUL e-cigarette mint flavored 5% nicotine e-liquid pods on adult ethanol consumption. Their findings indicated that adolescent nicotine vapor exposure (PND 30–46), voluntary ethanol consumption alone, and combination with nicotine exposure (i.e., nicotine vapor exposure, followed by voluntary ethanol consumption) did not alter ethanol intake or preference in adult SD [91]. IG adolescent ethanol exposure (PND 30–32; every 8 h for 2 days: 6 treatments total) also failed to alter ethanol and nicotine co-use during adulthood in SD rats [70].

However, several studies have indicated that co-exposure to ethanol and nicotine during adolescence results in distinct behavioral and neurochemical effects in adulthood that are not observed following adolescent exposure to ethanol or nicotine alone [92–94]. The effects of simultaneous exposure to ethanol and nicotine during adolescence have been reported to increase memory/learning deficits and enhance anxiety-like and drug-seeking behaviors in mice [93, 95, 96]. A recent study demonstrated that simultaneous IV self-administration of ethanol + nicotine during adolescence (PND 32–41) enhances ethanol reinforcement and intake in late adolescence and emerging adulthood in male rats (PND 48–65) [97]. A similar effect was not observed in male nor female rats when the same exposure and testing regimen occurred solely in adulthood (PND 90–99) [97]. Conversely, some studies have indicated that ethanol/nicotine co-exposure during adolescence (PND 30–45) results in certain adult alterations that parallel single drug exposure [98]. Taken together, these pre-clinical studies provide evidence that the effect of adolescent nicotine exposure or adolescent co-exposure to ethanol and nicotine on behavior is fairly complex, and further studies will be needed to determine how exposure, alone or in combination, during this developmental window affects use/abuse liability later in life.

## The effect of alcohol during adolescence on dopamine function

The mesocorticolimbic (MCL) dopaminergic (DAnergic) system is involved in processing the rewarding effects of natural reinforcers and drugs of abuse, and it undergoes significant developmental changes during adolescence [14]. For example, during mid-to-late adolescence, DA neurons in the ventral tegmental (VTA), that project to the nucleus accumbens (Acb), are firing at their highest rate, suggesting a pattern of DA overproduction and increased DA receptor expression throughout the circuit, which declines in adulthood [65, 99, 100]. Philpot et al. [101] provided further evidence that basal DA levels in Acb increased through developmental stages of preadolescent (PND 25; lowest DA levels), early adolescent (PND 35), and late adolescent (PND 45; highest DA levels) with a decline in young adulthood (PND 60).

Drugs of abuse effects on DA release are typically observed in the Acb. The Acb is divided into 2 distinct anatomical and functional structures (i.e., shell [AcbSh] and the core [AcbC]), and each plays a different role in reward and motivation. Reports have demonstrated that ethanol, nicotine, and other drugs of abuse preferentially increase DA release in AcbSh compared to AcbC [102]. Moreover, AcbSh receives more DA projections from the VTA than the AcbC [103]. The AcbSh plays a critical role in the reinforcing effects of rewards and spatial/contextual drug-seeking behavior, while AcbC is involved in the motivation to seek rewards and mediating cue-induced drug-seeking behavior [104].

The effects of adolescent ethanol exposure on DA release have been examined in both the AcbSh and AcbC. A consistent finding in the pre-clinical literature is that ethanol exposure during adolescence produces a persistent hyper-dopaminergic state, and this is observed in the AcbSh [65, 105–108]. Pascual et al. [65] reported that basal extracellular DA levels in the AcbSh, following AIE injections (3 g/kg/IP) in adolescence (exposure: PND 25–38; DA collection: PND 41) were higher than if the animals were animals pre-treated with ethanol during adulthood. Moreover, adolescent AIE exposure resulted in a higher expression of D1 and D2 receptors in adolescents compared to adult-exposed animals lending support to previous studies [65]. In a subsequent study, 4 days of repeated administration of low to moderate ethanol (0.5, 1.0, or 2.0 g/kg/IP) with the test days on PND 25 (preadolescent), PND 35 (early adolescent), and PND 45 (late adolescent) increased DA levels in the Acb in all the stages of adolescence [101]. However, the ethanol’s peak effects on DA decreased during pre-adolescence and early adolescence, following a challenge dose of ethanol, but was not altered in late adolescence or young adulthood [101]. These findings indicated that PND 35 and 45 appear to be a key developmental transition periods to the neuroadaptation of effects of repeated ethanol exposure [101]. In contrast to [65], this study found that young adulthood (PND 60) ethanol exposure also increases DA levels in Acb [101]. This difference between studies may be due to different ethanol exposure methods and doses of ethanol.

In another study, moderate ethanol exposure (1 g/kg, every other day) in adolescents (PND 30–50) reduced DA release in the AcbC following a challenge dose during adulthood, with the reduction becoming less robust as the abstinence period (7, 14, 49 days) between adolescent exposure and challenged ethanol dose test increased [109]. The authors finding suggested ethanol exposure starting during early adolescence, not during adulthood (PND 60–80), resulted in a decline in the responsiveness of DAnergic neurons in the AcbC to ethanol [109]. Collectively, these studies demonstrate adolescent ethanol exposure induces age-dependent effects on the MCL DAnergic system that may contribute to the increased risk of AUDs in adulthood.

Ethanol administration in adolescence has also been shown to increase basal DA levels in the AcbSh during adulthood [105, 106]. Injections of ethanol (0.75 g/kg/IP) for 21 consecutive days during adolescence (PND 30–50) produced a persistent increase in basal DA in the shell region of the Acb during adulthood (PND 70) compared to saline pre-treatment [106]. Further, the observed increase in DA was likely due to increases in efflux as there were no changes in DA reuptake [106]. In P rats, 24 h continuous voluntary ethanol consumption from PND 30 to PND 60 produced a prolonged increase (i.e., 2 h long) in the extracellular DA levels and increased DA uptake in Acb following ethanol challenge (2.5 g/kg/IP) in adulthood compared with saline-challenge [105]. The observed differences in the AIE induced increase in DA uptake between studies are possibly due to the use of different rat strains (P rats vs. SD). Research has found that P rats have an abnormal innate DA profile with lower DA and DA metabolites than their control, the non-preferring (NP) rats, which contributes to P rats' high ethanol drinking behavior [110].

The VTA is a heterogeneous structure. Intracranial self-administration studies, which elucidate specific neuroanatomical sites that support drug self-administration, have shown that posterior VTA (pVTA), but not the anterior VTA (aVTA), is a neuroanatomical site mediating the reinforcing actions of drug reinforcement (i.e., ethanol, cocaine, nicotine, delta9 tetrahydrocannabinol [111–115]). Furthermore, the reinforcing effects of pVTA require the activation of DAnergic neurons [116, 117]. Adolescent ethanol exposure has been shown to sensitize DA neurons in the pVTA as challenge doses of ethanol more readily enhance DA levels in adulthood [107, 108]. For example, P rats provided free access to ethanol during adolescence (PND 30–60), and Wistar exposed to an AIE regimen (PND 28–48), exhibited a leftward and upward shift of the dose-response curve when receiving microinjections of ethanol directly into the pVTA during adulthood. Together, these findings provide some evidence that ethanol exposure during adolescence may produce a hyper-dopaminergic system of ethanol that may, in part, underlie the biological basis for enhanced adult ethanol consumption.

## The effect of alcohol and nicotine on dopamine function

Ethanol and nicotine share some common mechanisms of action. For example, the reinforcing effects of nicotine are modulated via stimulation of nicotinic acetylcholine receptors (nAChR) receptors within the VTA [118, 119], and ethanol reinforcing effects are partially modulated by nAChR receptors [120–122]. For example, the antagonism of nAChR with mecamylamine, a non-selective nAChR antagonist, can reduce ethanol intake [123, 124] and nicotine-stimulated ethanol drinking [125, 126]. A limited number of pre-clinical studies have focused on the manner in which neuroadaptations that occur due to ethanol exposure during adolescence contribute to the co-use/abuse of drugs later in life. Recently, Waeiss et al. [127] observed that voluntary adolescent ethanol consumption (PND 30–60) increases the sensitivity (leftward shift in the dose-response curve) to the effects of nicotine within the MCL reward circuit (pVTA nicotine microinjection, DA release in AcbSh; 125). To date, however, the majority of studies have featured adult animals.

Combined peripheral injections of ethanol and nicotine, as well as a combined administration of low doses of ethanol peripherally and nicotine centrally (into VTA), have been shown to have an addictive effect on DA release in AcbSh of adult male Wistar rats [128, 129]. In adult P rats, acute exposure to ethanol and additional drugs (nicotine, cocaine, etc) results in unique, synergistic, or additive effects in various brain structures (e.g. [130, 131]). Chronic simultaneous ethanol and nicotine co-use results in unique adaptations in discrete brain regions that enhance drug reward in adult P rats [132, 133]. For example, microinjections of pharmacologically relevant doses of ethanol or nicotine directly into the pVTA induce DA release in the AcbSh [134]. Co-administration of subthreshold concentrations of ethanol and nicotine combined into the pVTA increases DA and glutamate release in the AcbSh, whereas the same subthreshold concentrations of each drug microinjected alone does not [130]. Furthermore, microinjections of ethanol + nicotine (but not ethanol or nicotine alone) into the pVTA altered the sensitivity of ethanol in the AcbSh and the expression of brain derived neurotrophic factor (BDNF) in the AcbSh (weeks following pVTA microinjections) [127]. Overall, the data indicate that co-administration of ethanol + nicotine differentially activates the mesolimbic DA system, which is not observed following the administration of the compounds individually.

## Potential pharmacotherapies to mitigate the effects of binge-like ethanol consumption during adolescence as it pertains to adult ethanol consumption

One of the primary goals of the pre-clinical adolescent ethanol research, is to develop useful strategies and/or pharmacotherapeutic treatments that are capable of counteracting the deleterious effects of binge-like ethanol consumption/exposure during adolescence and the subsequent increase in adult ethanol/drug use/abuse that may eventually be utilized to treat the clinical population. So far, the majority of research has focused on describing the biological consequences of adolescent ethanol exposure and there have been limited attempts to assess potential pharmacological interventions to counteract the behavioral and neurobiological consequences of adolescent binge-like ethanol exposure. Simply put, there are two approaches to counter the deleterious effects of binge-like ethanol exposure: reversal or prevention with the majority of effort focused on the latter. The few studies that have focused on “reversing” the effects of adolescent ethanol consumption/exposure have ‘focused on “correcting” the biological alterations produced by exposure to ethanol during the adolescent window.

Alterations in the central cholinergic system are implicated in nicotine and ethanol abuse [6, 135]. There are two classifications of cholinergic receptors: nicotinic and muscarinic. Nicotinic acetylcholine receptors (nAChRs) consist of 11 neuronal subunits, which are divided into 8 alpha subunits (*α2-α7*, *α9-α10*) and 3 beta subunits (*β2-β4*). Adolescent ethanol consumption/exposure has been reported to upregulate α7 nicotinic receptors, which are usually homomeric, during adulthood [127, 136]. Evidence also suggests that adolescent ethanol or nicotine exposure alone can lead to subsequent cholinergic dysfunction [6, 15, 137]. For example, choline acetyltransferase (ChAT), a cholinergic marker that is responsible for the biosynthesis of the neurotransmitter acetylcholine, is reduced in several brain regions following adolescent ethanol or adolescent nicotine exposure [6, 15, 137].

### Varenicline

This has led to an investigation of the effect of cholinergic compounds on adolescent ethanol exposure induced cholinergic deficits that persist well into adulthood [15, 137]. The FDA-approved smoking cessation aid Varenicline (i.e., Chantix), an α4β2 nAChR partial agonist, has a slower onset and longer duration on DA release compared to nicotine na d can block nicotine’s effect on DA release [138]. Varenicline has been reported to reduce ethanol consumption in adolescent C57BL/6J mice, after four 2 day DID sessions at PND 32–33, 36–37, 39–40, and 43–44, with Varenicline administered on the second of these 2 day sessions 30 min prior to ethanol access [139].

### Cholinesterase inhibitors

The FDA-approved cholinesterase inhibitors Donepezil and Galantamine administered during peri-adolescence to emerging adulthood (PND 69–72; PND 57–72) can reverse AIE (PND 30–48; PND 25–54) induced alteration or deficits (e.g., reversing AIE decreases in dendritic spine density or the persistent losses of cholinergic neuron markers) observed during adulthood in the hippocampus (i.e., learning and memory) and basal forebrain (i.e., behavioral control, attention, and other executive functions) [59, 137, 140]. Moreover, Galantamine administered during AIE exposure (PND 25–54) prevented the AIE induced deficits in the basal forebrain [137]. Interestingly, although Donepezil and Galantamine are both cholinesterase inhibitors, findings have demonstrated that Galantamine, but not Donepezil, increases the firing activity of DAnergic cells in the VTA [141]. Galantamine effect on DA through its allosteric potentiation of nAChRs [141]. Therefore, some of Galantamine’s blocking and reversing effects of AIE may partly be through the DAnergic system.

Galantamine effects also appear to involve the α7 nAChR mediating presynaptic facilitation of glutamate release [141]. In addition, the neuroprotective effects of Donepezil are mediated through α7 nAChR [142, 143]. Glutamatergic projections to MCL are involved in the development of drug-seeking and drug-taking behavior [144]. Moreover, excessive glutamate can induce excitotoxicity and loss of neurons [145]. The α7 nAChRs have a lower affinity for nicotine and are located presynaptically on glutamatergic terminals [146]. Thus, it is suggested that activation of α7 nAChR enhances glutamatergic excitatory drive and may promote DA release after the α4β2 receptors are desensitized [146]. Thus mediating the long-term effects of chronic nicotine exposure [147].

### α7 nAChR negative and positive allosteric modulator (NAM and PAM)

Administration of an α7 nAChR negative allosteric modulator (NAM) dehydronorketamine (DHNK) 2 hours before AIE exposure (PND 28–48) prevented the increase of ethanol consumption during acquisition and relapse drinking during adulthood in both male and female P rats [68]. A subsequent study reported that SB-277011-A, an α7 nAChR NAM and a D3 antagonist, could also suppress ethanol consumption during acquisition and relapse drinking in female P rats [69, 148]. In contrast, to α7 nAChR NAMs, activation of the α7 nAChR during adolescence appears to have the opposite effect on ethanol consumption in adulthood. Intermittent adolescent treatment (PND 29, 30, 33, 35, 36, and 37) with the α7 nAChR agonist AR-17779 increased the amount of ethanol consumed during acquisition and relapse during adulthood (PND 90+) in both male and female P rats [68]. Furthermore, co-infusion of α7 nAChR agonist + ethanol into pVTA increased extracellular DA release in AcbSh to a significantly greater extent than either treatment alone in male Wistar rats [69]. In addition, administration of α7 nAChR positive allosteric modulator (PAM) PNU- 120596 followed by low-dose ethanol (gavage, 2 mg/kg) in adolescents (PND 28–48) increased operant beer acquisition, extinction, and relapse drinking in adulthood in female Wistar rats [69]. However, PNU +2 mg/kg ethanol treatment during adolescence did not affect 24-h free-choice ethanol drinking of adult male Wistar rats [69].

Interestingly, AIE (PND 28–48) and peri-adolescent (PND 30–50) ethanol consumption did not alter glutamate release [108] or glutamate transporters [147] in Acb, respectively. However, adolescent nicotine consumption and ethanol + nicotine intake reduced glutamate transporter-1 (GLT-1), which reuptakes 90% of glutamate [145]. Therefore, further research is warranted to determine mechanisms that the α7 nAChR involvement with the glutamatergic system.

### Bupropion, Lobeline, and Cytisine

Other nicotinic cholinergic compounds have also been shown to have some effectiveness in reducing ethanol consumption in pre-clinical studies. For example, Bupropion is FDA-approved for smoking cessation as well as depression and seasonal affective disorders. It is an antagonist for α3β2, α4β2, and α7 nAChRs, with Bupropion being ∼50 and 12 times more effective in blocking α3β2 and α4β2 than α7nAChRs [149]. Bupropion is also a dual norepinephrine and DA reuptake inhibitor (cf., [149]). Bupropion effects on ethanol intake have varied with no effects on limited access (2 h) to ethanol in P rats [150] to reducing DID ethanol (2 h) intake in C57BL/J mice [151].

Lobeline is a non-selective antagonist nAChRs that can inhibit nicotine and ethanol DA release [152]. Lobeline also inhibits DA and vesicular monoamine transporters. Cytisine is partial agonist for α4β2 nAChR and a full agonist at β4 and α7 nAChR. Nicotine- and ethanol-induced extracellular DA release can be reduced by Cytisine [cf., 154]. Both compounds have been reported to reduce ethanol consumption in high-alcohol drinking models (i.e., C57BL/6J mice and high alcohol drinking line 2 rats [152–154]) as well as reduce ethanol-induced DA release [155]. However, no research has examined whether Bupropion, Lobeline, or Cytisine would effectively block or reverse the behavioral and neurobiological changes observed in adulthood following adolescent ethanol or ethanol+ nicotine exposure.

Collectively, these studies provide evidence that treatments targeting the nicotinic cholinergic system may represent viable pharmacotherapeutic compounds to ‘reverse’ the effects of adolescent ethanol exposure on ethanol-related neuropathology and drinking behaviors observed during adulthood. Moreover, it will be interesting to extend this line of research to investigate other nicotinic cholinergic compounds as well as examine the utility of these compounds to reverse the additive effects of adolescent ethanol + nicotine exposure on ethanol and/or co-use/abuse in adulthood.

## Conclusion

Pre-clinical and clinical research has indicated that adolescent alcohol and/or nicotine consumption/exposure can promote alcohol consumption during adulthood. The likelihood of observing this effect in pre-clinical research is increased if adolescent rats are exposed to biologically relevant levels of ethanol, with appropriate drinking protocols in adulthood, which produce pharmacologically relevant levels of ethanol intake. Recent experiments have indicated potential pharmacological targets that can reverse or prevent some of the persistent deleterious behavioral and neurobiological changes observed during adulthood following adolescent ethanol consumption/exposure. However, there is still a need to elucidate the mechanism and neural substrates that may contribute to the effectiveness of these cholinergic compounds. Pre-clinical research needs to be conducted to determine if the FDA-approved Donepezil, Galantamine, and Bupropion can attenuate adolescent alcohol and/or nicotine consumption/exposure ability to increase the risk of alcohol consumption during adulthood. Moreover, further research is still warranted to better understand adolescent co-use/abuse effects during adulthood, with a goal of developing novel efficacious pharmacotherapies to treat AUDs and co-use/abuse.
